# The academy and its journal: 45 years of editorial leadership in South African family medicine

**DOI:** 10.4102/safp.v68i2.6282

**Published:** 2026-03-31

**Authors:** Klaus B. von Pressentin

**Affiliations:** 1 Division of Family Medicine, Department of Family, Community and Emergency Care, Faculty of Health Sciences, University of Cape Town, Cape Town, South Africa

**Keywords:** family medicine, primary care, editorial leadership, academic publishing, journal evolution, capacity building, primary care research, scholarship, history of family medicine

## Abstract

Journals are living archives of their disciplines. For the *South African Family Practice* journal, its 45-year journey offers a unique lens on how family medicine in Southern Africa found its voice and evolved alongside global primary care scholarship. This Open Forum reflection traces the journal’s trajectory, from print and pharma-supported pages to a fully online, open-access platform grounded in ethical rigour and robust peer review. Drawing on archival insights and a conversation with Prof. Pierre de Villiers, who continued Prof. Sam Fehrsen’s editorial legacy, the overview highlights how editorial independence, methodological diversity and capacity building have shaped the journal as a trusted voice for family medicine and primary care. This narrative describes the journal’s role in enhancing the discipline’s evidence base and scholarly identity over 45 years. The evolution of the journal offers lessons for strengthening primary care scholarship in Africa and other regions.

**Contribution:**

Four decades of editorial leadership of the *South African Family Practice* have significantly shaped the journal’s identity, integrity and reputation in primary care across Southern Africa and abroad. Key transitions supporting inclusive scholarly development are highlighted.

## Introduction

The *South African Family Practice (SAFP)* journal has developed alongside the discipline of family medicine in South Africa. At 45 years old, the journal offers a chance to reflect on its growth and impact, from a modest publication for busy general practitioners to a comprehensive, entirely online, open-access scholarly journal with a diverse research focus and a strong commitment to social responsibility and relevance to Southern African primary care.

Scholarly journals serve more than by simply disseminating research; they also act as a testing ground for shaping a young discipline’s identity, providing a platform for dialogue and the development of evidence. Geyman argued that journals provide a ‘window to an evolving academic discipline’, encouraging critical inquiry and collaboration.^1^ In its early stages, family medicine faced scepticism about its intellectual worth and struggled to establish a distinct knowledge base separate from other specialities. As Gotler notes, the early neglect of research left ‘unfinished business’ for the discipline, affecting its ability to lead health system transformation today.^2^ To inform this iterative process of identity formation for our discipline, family medicine and primary care providers should be involved in research, given the unique settings in which they work and their scope of practice. Primary care providers have ‘unique expertise and unique challenges’ that can inform their engagement with existing evidence, as well as generate novel questions to inform new lines of inquiry.^3^

Howard Stein, paraphrasing Erik Erikson, observed that identity formation reflects an understanding of who and what one is, both internally and through interaction, and rarely follows a linear path.^4^ Instead, it is characterised by periods of discord and crisis that foster opportunities for growth. Over the past century, the global field of family medicine has undergone similar changes influenced by both internal developments and external factors. Disciplines may evolve, split or re-emerge, as demonstrated by the transformation of family medicine from its initial roots, a process Ian McWhinney examined in his foundational work.^5^

**FIGURE 1:**
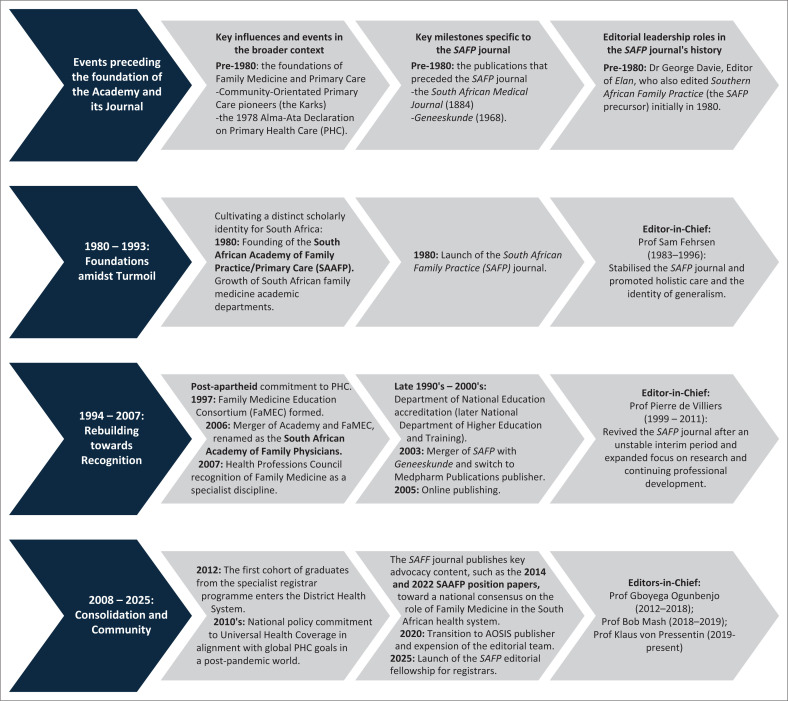
Timeline of the *South African Family Practice* journal’s evolution.

This retrospective overview aims to capture the *SAFP*’s role in the evolution of South African family medicine as an emerging academic discipline. Founded in 1980 alongside the South African Academy of Family Physicians (SAAFP), the journal marked a pivotal milestone in shaping the discipline’s identity. By adopting a life-course approach, this overview aims to capture the events, intellectual contributions and strategic perspectives of past editors who have shaped the journal’s evolution from its origins to the present day. As two sides of the same coin, this intertwined history of the SAAFP and its journal demonstrates an ability to turn moments of discord and crisis into opportunities for dialogue and growth. To understand this trajectory of growth, we need to revisit the journal’s roots and the forces that shaped its identity. A timeline of the journal’s first 45 years (Figure 1) situates its development within broader historical contexts in Primary Health Care (PHC): the emergence of community-orientated primary care (COPC) in mid-20th-century South Africa, its links with international family medicine and general practice and the impact of the 1978 Alma-Ata Declaration from the International Conference on PHC, which defined health as a fundamental human right and established PHC as the key to achieving ‘Health for All’.

In a ‘*SAFP* at 45’ panel discussion at the 2025 27th National Family Practitioners Congress, the current editorial team shared reflections on past and present developments to stimulate dialogue with congress participants and elicit thoughts on future directions.^6^ The methods used to inform this overview involved incorporating the panel discussion outputs and a review of key publications. The overview was further refined through an interview with a former editor. As the current Editor-in-Chief, I am aware that my own perspective may have influenced the writing of this overview. This deep dive into the *SAFP* origin story made me even more fond of ‘the journal’, as previous and current editors refer to it, reflecting ‘a term of endearment, respect, and recognition of the important role it played in the development of the discipline of family medicine’.^7^ By tracing its evolution across the preceding decades, I hope this narrative review offers lessons for strengthening primary care scholarship in Africa and other regions.

## Historical foundations

One might ask how academic primary care evolved and what publishing platforms were available in our context. The *South African Medical Journal (SAMJ)*, first published in 1884, was the country’s earliest medical periodical and, for decades, the primary scholarly outlet for doctors.^8,9^ It merged in 1926 with the *South African Medical Record* (founded in 1903) and the *Transvaal Medical Journal* (founded in 1906), becoming the official journal of the Medical Association of South Africa. For much of the 20th century, *SAMJ* dominated the medical publishing landscape. In 1968, *Geneeskunde* was launched to cater to Afrikaans-speaking practitioners and remained a key alternative for general practice and family medicine scholarship until its eventual incorporation into the *SAFP*. These journals provided continuity for professional discourse long before family medicine achieved speciality status, shaping the intellectual environment into which our journal emerged.

Importantly, before the birth of the *SAFP* journal in 1980, the foundations of academic family medicine had already been laid. The development of COPC provided a local intellectual tradition emphasising population–based strategies within defined communities. Pioneered by Sidney and Emily Kark at Pholela in the 1940s, COPC integrated promotive, preventive and curative care through community health centres, influencing the 1944 Gluckman Commission’s vision for a national health service.^10^ This innovation extended into medical education when the University of Natal’s Faculty of Medicine established the Department of Social, Preventive and Family Medicine in 1956, with Kark as its first head, and embedded COPC principles into clinical training through the Institute of Family and Community Health.^11,12^ These initiatives represented an early attempt to build family medicine as an academic discipline, but no evidence was found of efforts to develop a dedicated academic family practice scholarly journal during the pre–apartheid era. Political resistance and the dismantling of these structures in the late 1950s curtailed the growth of the discipline, delaying formal academic consolidation until decades later.^10,12^

Early professional communication and continuing education in general practice took the form of newsletters that included original articles and clinical updates. In 1958, initial connections with the United Kingdom’s Royal College of General Practitioners were established in South Africa through regional faculties in the Cape of Good Hope and the Witwatersrand (the Orange Free State, Eastern Cape, and Natal faculties followed shortly).^12,13,14^ A significant milestone was the appointment of Howard Botha as the first professor of family medicine at the University of Pretoria in 1967.^15^ The South African College of General Practitioners was founded on 1 June 1969. In 1970, it merged with the College of Physicians, Surgeons, and Gynaecologists to form a single College of Medicine of South Africa (CMSA). After the merger, the College of General Practitioners became the Faculty of General Practice within the CMSA. By joining the CMSA, the College of General Practitioners (now a CMSA Faculty) acquired the infrastructure to organise fellowship, diploma, and certificate examinations for general practitioners. (In April 1998, after transforming faculties into colleges, the parent organisation was renamed the Colleges of Medicine of South Africa.) In the Cape of Good Hope region, Dr Seymour Dubb edited the Faculty of General Practice newsletter ‘with wit and distinction’, while in the North, Dr George Davie edited *Elan*, which evolved from a newsletter into a mini–journal.^16^ After the College of General Practitioners joined the CMSA in 1970, these two newsletters merged, and *Elan* became the official journal of the Faculty of General Practice under Davie’s editorship. This transition marked an essential step towards establishing a scholarly identity for general practice in South Africa, paving the way for subsequent developments, sincluding the *SAFP*, which grew out of the successor to *Elan*, the *Southern African Journal of Family Practice*.^12,14,16,17^

Tensions over the Faculty of General Practice’s autonomy within the CMSA began in August 1980, ultimately leading to the decision to establish and register a new independent organisation. These issues had been brewing since shortly after the first General Practice Congress in Johannesburg in 1978. As Basil Jaffe reflected, the congress was:

[*A*] resounding success and a revelation to us all the talent that exists in our own ranks. It generated a great deal of enthusiasm that helped us through the troubled times that lay ahead in our relationship with the College of Medicine.^13^

The conflict came to a head when the faculty leadership and the CMSA Council clashed over a clinical trial of a new beta-blocker for hypertension. The Council argued that the trial was promotional and should not appear under the CMSA’s name although some members disagreed. This disagreement exposed deeper structural concerns: limited voting rights, restricted control over examinations, research, and publications and an overall lack of autonomy.^13^

Prolonged tension consumed considerable time and energy, making the situation untenable. After extensive deliberation, the faculty resolved to form an independent body open to practitioners who shared these objectives:

- Raise and maintain the standard of general practice in South Africa.
- Achieve recognition of family medicine as an academic discipline in all spheres of medical education and practice.
- Help provide a system of primary care for all people in this country.

The new body was named the South African Academy of Family Practice and Primary Care (SAAFP). This combined name reflected two intentions: to align with the 1978 Alma-Ata Declaration, which emphasised the importance of PHC globally, and to assert that family medicine is integral to primary care delivery, while recognising that other disciplines also contribute. The SAAFP affirmed that the principles of family medicine remain consistent across geography and socio-economic contexts and embraced the Alma-Ata WHO-UNICEF definition of PHC. The plan to establish the SAAFP was enthusiastically received when announced during the keynote address at the second GP Congress in Cape Town (March 1980). The SAAFP was officially registered on 11 August 1980. Inaugural meetings were held nationwide to explain its objectives, structure and functions, and plans were made for national, regional and small group structures to encourage broad participation.

Despite some opposition, the CMSA Council supported the Academy’s establishment, approved the transfer of Faculty funds and allowed continuation of activities. The faculty decided to stay within the CMSA, which would continue to serve as the examining body for family medicine and its membership examination. (This Faculty of General Practice later became the College of Family Practitioners and eventually the College of Family Physicians.)

The first Academy Council elections took place in 1981, ensuring regional representation and diversity. In his address at the third GP Congress in Sun City from 09 August 1982 to 12 August 1982, the first SAAFP president, Dr Boz Fehler, reflected on the past, present and future of general practice in South Africa. He noted that general practice in South Africa has evolved from a marginalised role to a recognised cornerstone of PHC, and its future depends on committed practitioner engagement, robust education and collaborative efforts to strengthen standards and access. He called on the government to fund ‘these provisions and establish departments of Primary Care at all medical schools in the Republic’ as, among other factors, ‘ongoing research and evolution [are] essential for the future development of Family Medicine in South Africa’.^18^

A significant portion of the SAAFP’s early efforts focused on establishing academic family medicine departments to provide adequate training and education. By 1977, some nascent departments were already functioning at the University of Pretoria and the Medical University of Southern Africa (MEDUNSA, now Sefako Makgatho Health Sciences University) and by 1978, the University of the Orange Free State.^19^ In the 1980s, through its Academy Education Committee, the SAAFP addressed the shortage of doctors in rural areas through vocational training. In 1997, the Family Medicine Education Consortium (FaMEC) was formed, bringing together the now eight existing academic departments of family medicine in South Africa to form a unified academic organisation of family practitioners. This consortium focused on functions that include performing educational activities, setting standards and conducting postgraduate examinations.^19,20^

Circling back to 1980, we pick up the trail again of identifying the point of inception for our journal. Before the Academy was established, the *Southern African Family Practice* (note the different spelling) was a commercial journal owned by Thomson Publications SA, with its first issue published in January 1980. At the Academy’s establishment, it was agreed that the journal would become its official publication under the editorship of Dr George Davie (the previous editor of *Elan*). Davie’s editorial leadership and reputation for shaping general practice journalism – described as bringing an ‘inimitable style and flavour’ and often being ‘provocative with tongue in cheek’ – made him the natural choice to guide the Academy’s new journal, marking the shift from regional newsletters to a national platform for family medicine scholarship.^16^

In January 1981, the masthead of the journal started to carry the words, ‘Official Journal of the South African Academy of Family Practice/Primary Care’.^17^ Thomson Publications SA granted the Academy the first option to buy if it stopped publishing, which subsequently occurred in 1983. The Academy became its publisher, registered the name *South African Family Practice* (as the journal is still known today) and appointed Prof. Sam Fehrsen as editor. Fehrsen was selected for his academic stature as Head of Family Medicine at MEDUNSA and for his success in creating a department closely linked to community-based primary care, bringing both academic and administrative expertise to strengthen the journal’s role as a forum for a diverse membership.^13,16^ The first issue under the Academy’s ownership, featuring a new format and image, was released in January 1984. For the first 2 years, the Academy also had a short-lived medical newspaper, *Academy Post*, a commercial venture by Keeble-Prins, which helped publicise the Academy’s launch but eventually closed.^13^

In summary, the Academy and its journal were launched in 1980 as ‘twins’, and their intertwined paths would prove vital for consolidating the discipline’s philosophy and practice in South Africa, even during social and political upheavals. The international commitment for PHC at Alma-Ata (1978) occurred when general practitioners and missionary doctors played key roles in developing South African primary care with little government policy backing. A more substantial policy commitment to PHC was introduced only with the advent of democracy in South Africa in 1994, with the district health system designated as the foundation of healthcare.^21^

## Prof. Sam Fehrsen: Establishing the journal’s philosophy (1983–1996)

Prof. Sam Fehrsen’s leadership across 13 years established a clear philosophical direction for the journal. In a 1996 interview, Fehrsen described the journal’s role as ‘a means of helping to build the discipline’ and ‘moving away from the mechanistic view of health and illness’, resulting in ‘very dehumanising experiences for both patients and doctors’ towards ‘the holistic view of the general practitioner’s work’.^22^ He warned that unless ‘we start thinking differently about illness’, the family physician would find it difficult to secure a future within ‘the broader medical scheme of things’.^22^ This philosophy validated person–centred care, continuity and the primacy of context as scholarly themes and promoted qualitative inquiry alongside trials and other conventional research designs.

Under his editorship, the *SAFP* became the Academy’s official journal, publishing original articles, congress papers, clinical reviews, updates and regular columns such as Human Behaviour, Clinical Corner and Journal Club, along with regional news and event listings. Fehrsen facilitated initiatives such as writer workshops to boost contributions, and two notable contributors, Drs Russell Kirkby and Chris Ellis, added significant value through their columns and editorial work. The journal was widely read and primarily sustained by pharmaceutical advertising.

The journal was also linked with other Academy publications.^13^ In 1982, a *South African Manual for General Practice* (the precursor of the well-known *South African Family Practice Manual*) was made available to members through Merck, Sharp and Dohme (MSD), which had purchased the publishing rights. Initially developed by a New Zealand family doctor, it was adapted for South Africa by Prof. GJ Pistorius, who also led an editorial board producing updated chapter supplements distributed by MSD. In 1989, MSD donated the Manual to the Academy, after which updated chapters were published quarterly as supplements to the *SAFP*, ensuring that each of the 12 chapters was revised every 3 years. Dr M Teichler (MEDUNSA) edited these supplements, which were republished in book form in 1995. Later, Dr G Ogunbanjo, also from MEDUNSA and later to become the continuing professional development (CPD) editor and Editor-in-Chief of the *SAFP*, began producing a revised edition. From its early years, the Academy also produced educational booklets – starting with an information brochure by Dr B Jaffe, vocational training and health education booklets by Dr J Smith and a hypertension update by the Southern Transvaal Region in 1983.^23^ Between 1984 and 1988, the *SAFP* published six educational booklets as journal supplements.

During the first decade of ownership, the journal and Academy thrived, enabling the Academy to acquire its own property, Academy House in Johannesburg.^17^ However, fortunes later declined because of over-reliance on pharmaceutical advertising for revenue. The 1996 interview with Fehrsen also echoes the journal’s modest, resource–limited beginnings (reflecting both constraints and a strong sense of purpose) and the conviction that GPs need ‘no longer apologise’ for not specialising in organ–system subspecialties, as their contribution lies at the interface between practitioner and patient.^22^ Fehrsen envisioned the journal as a platform for a paradigm shift in South African medicine and healthcare. His early editorials were known to stimulate debate among the journal’s readership. As an example, his 1986 editorial ‘GPs have nothing to offer’ used a statement by a colleague of his that ‘There is nothing a general practitioner can teach an undergraduate student that a specialist cannot teach better’.^24^ He mused that he and his colleague could not agree on a starting point for this fallacy and posed, ‘Is this perhaps the reason why some of our universities have no, or merely embryonic departments of general practice?’

His emphasis on a holistic and relational approach echoes later editorials that would reintroduce family medicine as a ‘counterculture’ within the dominant technocentric tendencies of modern healthcare, and his work as a thought leader echoed the international reflections by intellectuals such as GG Stephens.^25^ This ethos recognises rigour and relationship as mutually reinforcing rather than mutually exclusive. The journal’s early focus on the idea that doctor and patient experiences deserve rigorous study laid the groundwork for a broader, evidence-based approach to primary care.

## Interregnum and the risks of discontinuity (1996–1998)

Following Fehrsen’s departure in 1996, the journal experienced instability. Caretaker editors, irregular publication schedules and a diminished publishing cadence threatened its continuity during this retrospectively viewed ‘in-between time’ (interregnum); in 1998, only three issues were published (it had been a monthly journal). The situation threatened to limit the journal’s influence as family medicine was shaping its identity within a transforming health system, especially given the post-apartheid government’s focus on the district health system. The transition to democracy emphasised PHC, creating an ideal environment for research on family medicine’s role in the public health system.^21^ This period exemplifies a common challenge in scholarly publishing: editorial stability and a consistent schedule are not just operational concerns but essential for maintaining a credible platform.^7^

## Prof. Pierre de Villiers: Modernising the journal (1999–2011)

Prof. Pierre de Villiers started his 13-year term as editor in 1999 with a clear mandate: restore stability, safeguard independence and establish this journal as a reputable, versatile platform serving both practitioners and academics. His retrospective account, including notes from a recent interview as illustrated in Box 1, up to his final editorial in 2012, highlighted three key strategic turning points: being indexed and accredited by the Department of Higher Education and Training, which established the journal as a recognised publication outlet and allowed universities to receive publication subsidies; the 2003 merger with *Geneeskunde*, which strengthened the CPD section and broadened the journal’s readership; and the 2005 transition to a fully online ‘virtual publishing office’, hailed as the first among South African scholarly journals.^26^ This move streamlined workflow, improved indexing and archiving and foreshadowed later efforts towards wider open access.^7^ De Villiers’s reflections stress the importance of editorial independence, strengthening peer review and a practical commitment to serving the discipline’s dual role: CPD for practitioners and peer-reviewed research for the academic community. These journal activities also aligned with broader developments when the Academy and FaMEC merged to form the ‘new Academy’ in 2006, and the Academy was renamed the South African Academy of Family Physicians, which was reorientated in 2007 to serve as the national professional body representing the new speciality of family medicine.^27,28,29,30,31^

BOX 1 Interview insights: Prof. Pierre de Villiers on modernising the journal and expanding its academic influence in supporting the emerging speciality.

In our online conversation on 22 October 2025, we reflected on Prof. Pierre de Villiers’ experience as Editor-in-Chief, around 14 years after he completed his term. He assumed the editorship amid instability; he described the journal as ‘almost dead’, following a period when caretaker and interim editors took over after Prof. Sam Fehrsen stepped down in 1996.

Tracing back to the journal’s origins as a voice for private general practitioners, de Villiers noted that these beginnings reflected the Academy’s early roots in the private sector. ‘The SAAFP and the journal shared a journey’, he explained. ‘In the early years, the Academy had strong links with the private sector, and many readers were GPs in private practice. The pharmaceutical industry strongly supported journals, but by the time I came on board, that support had waned. Private GPs were too busy, and the academic footprint was too small’. The challenges were significant: a shrinking contributor base, irregular publication schedules and financial uncertainty. ‘One of the early tensions was reliance on pharmaceutical advertising’, he recalled. ‘We had to draw a clear line between advertorials and genuine editorial content. That centred on ethics, transparency, and peer review’.

Having recently entered academia, de Villiers accepted the role in 1999. ‘I enjoyed the challenge’, he said. ‘I had only been in academia for a few years and was focused on establishing training programmes. We had to establish the discipline’s research foundation. I even pursued further postgraduate training in epidemiology and was the first doctoral graduate at Stellenbosch University who came from family medicine. I encouraged other academic departments to help grow the research presence in the journal, publish MFamMed student research, and increase conference presentations’. His vision rested on two pillars: ‘Acknowledging the speciality status of the discipline with registrar training programmes, and building the body of knowledge to differentiate family medicine from other disciplines. That was the journey we walked’.

Prof. de Villiers recognised that sustainability depended on expanding the journal’s reach beyond a small community of SAAFP members and GPs. ‘Who was reading the journal? Mostly GPs in private practice. This paved the way for internationalisation. The online publication was key and aligned with the open-access movement. The *SAFP* became the first South African academic journal to be online, and a website was developed with the help of an Information Technology student, Dewet Diener. Medpharm was the publisher of the printed edition, and I generated the online version’. He highlights this milestone as transformative: ‘The move to open access was, for me, the major game-changer – the outstanding event around 2004 or 2005. Online reach brought new communities into the conversation. We saw stronger engagement from young family physicians and departments across the country and continent’. He adds: ‘Indexing in 2004 and the integration with *Geneeskunde* were important steps, and there was an attempt to establish a unified, strong journal for the discipline. However, these were not the game-changers – the move to online and open access was’. A few years later, JP van Niekerk, the editor of the *SAMJ*, reached out to them after discovering our journal’s online option, which also helped facilitate moving the *SAMJ* online.

Under his leadership, the journal also became a platform for debate on critical issues: vocational training versus speciality recognition, rural medicine as a separate discipline and the way to keep public- and private-sector doctors united. ‘It was always uneasy’, he admitted. ‘The philosophy was to establish a basic qualification in family medicine which would equip the graduate to work in any part of the country – be it an urban GP practice in Sea Point or a public sector district hospital in Beaufort West’. The career medical officer and the GP could benefit from a diploma in family medicine, which may be required for independent practice, rather than vocational training. The aim was to establish a minimum standard for independent practice, particularly in primary care. The diploma could serve this purpose, and this needs to be revisited now with the discussions on establishing the National Health Insurance.

Prof. de Villiers also played a pivotal role in strengthening academic collaboration. ‘We established FaMEC as the vehicle under the umbrella of SAAFP’, he explained. ‘It brought together academic departments and created one umbrella organisation. FaMEC helped unify educational standards, examinations, and research priorities. The journal became the avenue for building the body of knowledge for our discipline and was later supported by the launch of the regional journal, the *African Journal of PHC and Family Medicine* (*PHCFM)*. This created a snowball effect – postgraduate programmes matured, PhD pathways expanded, and a growing nucleus of researchers emerged’.

His advice to future editors remains clear: ‘Protect independence, adhere to rigorous peer review, and serve the entire discipline, both public and private. Lessons learned from those who manage the organisation need to be thoughtfully considered. Without strong governance and a clear business model, even the best editorial vision cannot survive’.

SAAFP, South African Academy of Family Physicians; SAFP, *South African Family Practice*; GP, general practitioner; FaMEC, Family Medicine Education Consortium.

In a reflective interview (Box 1), we revisited early tensions, including the challenge of clearly distinguishing between editorial content and commercial advertising. We highlighted how the shift online, coupled with open access, expanded reach and relevance, attracting a wider readership beyond academic centres and fostering practice-oriented dialogue. The editorial stance was never to choose between CPD and scholarship but to integrate them into a coherent identity for a primary care journal.^7^ De Villiers ended his farewell editorial by observing that, although the journal had achieved much, it would continue to need ‘steady hands’ because ‘the waters of scholarly publication and medical politics can get rough at times’, a reminder that independence and consistency are ongoing priorities.^7^

## Prof. Gboyega Ogunbanjo: Expansion and consolidation (2012–2018)

In his first editorial in 2012, Prof. Gboyega Ogunbanjo described his journey from *SAFP* associate editor (6 years) with a 3-year overlap as Editor-in-Chief of the first *African Journal of PHC and Family Medicine (PHCFM)* until 2011.^32^ In 2012, a ‘change of the guard’ saw Prof. Pierre de Villiers take over as *PHCFM* Editor-in-Chief and Prof. Ogunbanjo take over as *SAFP* Editor-in-Chief. He framed Prof. De Villiers’ wish of ‘Bon voyage’ in his final editorial as commendable, ‘but for me, it is “Aluta continua”’. During his tenure, Prof. Ogunbanjo’s vision was to have the SAFP journal indexed in ‘Index Medicus’ to complement the existing indexing in international and regional databases. He also committed to ensuring free online access to the journal’s archive.

Under Prof. Ogunbanjo’s tenure, the journal broadened its editorial scope by regularly publishing thematic editorials that addressed system–level issues, including the implementation of the National Health Insurance, predatory publishing, non–communicable diseases and environmental health factors.^33,34,35,36,37^ Ogunbanjo had served as CPD editor for several years alongside De Villiers before taking on the role of Editor-in-Chief. His editorial leadership ensured that the editorials and open-forum submissions strengthened the journal’s role in public discourse and advocacy within the SAAFP community and beyond, linking clinical realities with policy discussions and ethical concerns.^6^ Key publications during his tenure included the first national position paper for South Africa on the contribution of family physicians to district health services, prepared for the National Department of Health following a roundtable meeting with representatives of the discipline of Family Medicine of the SAAFP and the College of Family Physicians of South Africa.^38^

As part of his academic citizenship, he played a significant role in transforming family medicine into a full speciality in South Africa.^39^ He was a leading figure in family medicine at the national and subcontinental levels, serving as Vice-President of the SAAFP and Honorary Registrar: Examinations of the Colleges of Medicine of South Africa (CMSA). He held various editorial roles, including Editor-in-Chief of the *SAFP*, Editor of the *Transactions* journal of CMSA and as an advisor to several journals. He frequently spoke at national, African and global family medicine and PHC conferences.

Sadly, Prof. Ogunbanjo passed away in August 2019, yet his legacy will continue through his many scholarly outputs and the lives he has touched. A transitional period under Prof. Bob Mash (2018–2019) preserved editorial continuity and prepared for my appointment as the new Editor-in-Chief in March 2019.^40^ A reflection on our current editorial team’s experience is shared in an editorial introducing the 45-year special collection.^41^

### Limitations

Assessing historical documents as secondary sources requires careful consideration of their limitations. These accounts often mirror the dominant sociopolitical ideologies and institutional agendas of their time, which can introduce biases and exclude marginalised perspectives. Changes in language and terminology over time increase the likelihood of misinterpretation. Additionally, many documents were created for advocacy rather than impartiality, potentially affecting their reliability. Applying contemporary values to interpret historical events can lead to a distorted understanding. Critical evaluation involves contextualising sources within their original period and cross-referencing with diverse evidence. This approach is essential in the history of family medicine, in which paradigms and sociopolitical influences shape narratives. In my review, I analysed previous editorial titles to identify common themes, ensured accuracy by cross-checking sources and interviewing a former editor and aimed to deliver a credible account. Although interviewing other former editors would have been very helpful, it was unfortunately not feasible, and I had to rely on published sources.

### Recommendations

Future research should explore the history of family medicine and its scholarly publications, critically engaging with archives and other primary sources that may be biased and thus limited.^42^ Through archival research, thematic analysis and oral histories, scholars must also investigate how sociopolitical shifts and paradigm changes influence disciplinary identity. Examining global trends alongside local realities can reveal how family medicine adapts within evolving health systems. Digital preservation and collaboration are crucial for transparency and accessibility. Collaborating with scholars from other disciplines will support interdisciplinary research. The journal’s complete archive is now accessible online thanks to our publisher’s efforts, which will support the proposed research activities.

## Conclusion

The history of the *SAFP* journal shows consistent dedication and quick adaptation during periods of discord and crisis. From Fehrsen’s philosophical foundations to de Villiers’s strategic modernisation and Ogunbanjo’s calls to address systemic issues, the journal has consistently aligned its strategy with the evolving needs of primary care teams and their communities. The main lesson from 45 years is that trustworthy scholarship’s core principles are clear and attainable, yet upholding these principles demands ‘steady hands’, vision and renewed dedication. As South Africa moves towards universal health coverage, the journal’s history offers guidance on sustaining scholarly integrity amid systemic change.
